# Kimura’s disease with membranoproliferative glomerulonephritis: a case report with literature review

**DOI:** 10.1080/0886022X.2019.1584115

**Published:** 2019-03-26

**Authors:** Sensen Su, Xin Chen, Jia Li, Jinyu Yu, Li Zhang

**Affiliations:** a Department of Nephrology, the First Hospital of Jilin University, Changchun, China;; b Department of Digestive Endoscopy, the Second Hospital of Jilin University, Changchun, China

**Keywords:** Kimura's disease, membranoproliferative glomerulonephritis, nephrotic syndrome, prednisone

## Abstract

**Background:** Kimura's disease is a rare disease and its etiology is still unclear. Here we reported a case with lymphadenopathy complicated with secondary membranoproliferative glomerulonephritis.

**Case presentation:** A 46-year-old Chinese man presented with bilateral tumor-like nodules over his neck during the past 6 months and developed edema for 15 days. His blood pressure was 145/90 mmHg, multiple 1 × 1 cm masses were found over bilateral post-auricular and submandibular areas, along with trace edema of the lower extremities. Laboratory data showed an increased peripheral eosinophil count at 3.66 × 10^9^/L (50% of total leukocytes), with a 24-hour urine total protein of 8 g and a serum albumin of 19 g/L, and serum IgE of 2930 IU/ml (<100 IU/ml). The patient underwent renal biopsy, which revealed membranoproliferative glomerulonephritis with eosinophilic infiltration of the interstitium. Lymph node biopsy showed eosinophilic lymphoid follicular granuloma. Bone marrow biopsy showed no abnormalities. A diagnosis of Kimura's disease was then established. We started him on prednisone 60 mg/day (1 mg/kg), and tapered the dose to 55 mg/day 2 months later, followed by a gradual reduction of 2.5 mg every 2 weeks. Valsartan was given for blood pressure control. His neck nodules shrank after 2 weeks of treatment and complete renal remission was achieved 3 months later. No relapse occurred after follow-up for 31 months.

**Conclusion:** Kimura's disease can present with bilateral neck nodules and nephrotic syndrome (membranoproliferative glomerulonephritis), and prednisone can be a suitable choice of treatment.

## Background

Kimura’s disease was first described by Chinese surgeon Kimm in 1937 and was coined in 1948 according to Japanese pathologist Kimura. It is a rare disease with unknown etiology. Its manifestations frequently involve subcutaneous tumor-like nodules over the head and neck areas, accompanied by lymphadenopathy and occasionally major salivary gland pathology. It is not uncommon for patients to exhibit peripheral eosinophilia and increased serum IgE. Renal involvement often occurs. Although Kimura’s disease is benign in nature, it tends to recur. Existing cases of Kimura’s disease mostly come from Asian countries, including Japan, China, and India, with a male to female ratio about 3.5:1 [1]. In a review by Yamada et al. [2], they found that renal involvement occurred in 12% patients (21/175), of which 62% (13/21) presented with nephrotic syndrome. Chen et al. [3] summarized 29 cases with renal involvement from China and suggested that the most common clinical features were proteinuria, hypertension, and microscopic hematuria. During pathologic examination, light microscopy could manifest as mesangio-proliferative glomerulonephritis (*n* = 14), minimal change disease (*n* = 8), focal segmental glomerulosclerosis (*n* = 3), membranous nephropathy (*n* = 2), membranoproliferative glomerulonephritis (MPGN; *n* = 1), and acute tubular necrosis (*n* = 1). We herein reported a case with initial presentation of tumor-like nodules over the neck area and nephrotic syndrome which was finally diagnosed as MPGN.

## Case presentation

A 46-year-old Chinese man was admitted with the initial presentations of bilateral tumor-like nodules over the neck during the past 6 months and leg edema for 15 days. The neck nodules enlarged during 6 months and were painless without pruritus or dermatitis. He also had a body weight loss of 10 kg during the past 6 months, without symptoms such as fever, cough, diarrhea, or oliguria. On examination, multiple 1 × 1 cm hard and movable masses were found over bilateral post-auricular and submandibular areas, with intact overlying skin and a sharp boundary. His blood pressure was 145/90 mmHg. Trace edema was found in his lower extremities, but other physical examination results were normal.

Complete blood count showed an increased eosinophilia count at 3.66 × 10^9^/L (50% total leukocytes). His serum creatinine was normal, with a 24-h urine total protein of 8 g and a serum albumin of 19 g/L (normal, 40–55 g/L). Serum IgE was elevated, at 2930 IU/ml (<100 IU/ml), while IgG (3.61 g/L; normal, 7.0–16.0 g/L) and C3 (0.75 g/L; normal, 0.9–1.8 g/L) were decreased. IgG4 was within normal range. His anti-nuclear antibody and anti-neutrophil cellular antibody were normal, and hepatitis B virus surface antigen, hepatitis C virus, and human immunodeficiency virus serology were all negative. An ultrasound examination of tumor-like nodules revealed swelling lymph nodes located over bilateral neck and supraclavicular areas, while renal ultrasound, chest X-ray, and electrocardiogram were all normal.

**Figure 1.**
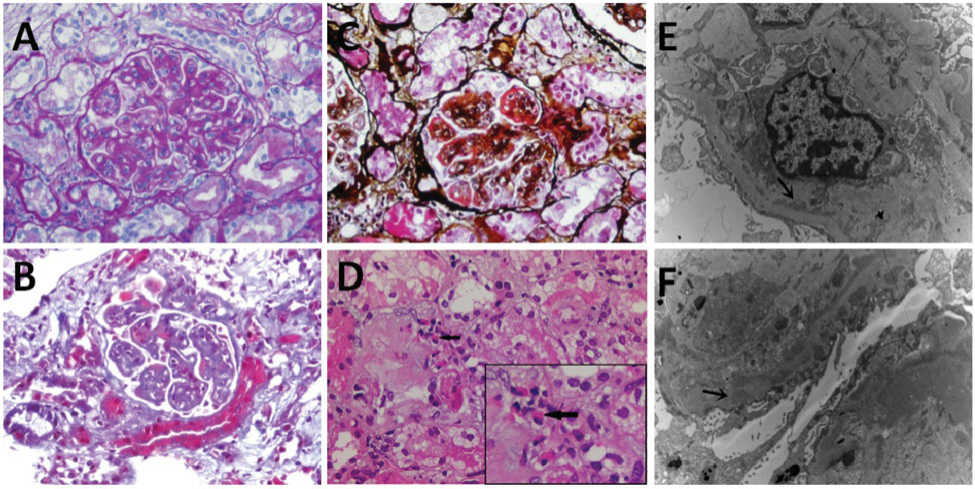
Renal specimen showed membranoproliferative glomerulonephritis (A, B, C) along with eosinophils infiltration in the interstitium (D: arrow). Electron microscope showed electron dense deposit in endodermic (E: arrow) and subepithelial (F: arrow) region. (A: PAS × 400; B: Masson × 400; C: PASM + MASSON × 400; D: HE × 400; E&F: EM × 10000).

The patient then underwent renal biopsy. Renal biopsy revealed MPGN with eosinophilic infiltration of the interstitium. Light microscopy of 15 examined glomeruli showed mesangial proliferation involving all glomeruli; mesangial matrix inserting into capillary loops; endothelial cells showing segmental swelling with hyperplasia; thickening glomerular basement membrane with diffuse dual-track sign; protein deposition under endothelial cells; and platinum loop sign (Figure 1). Some of the glomeruli were infiltrated by inflammatory cells. Renal tubular epithelia showed vacuolar degeneration, with eosinophilic infiltrations noted over the interstitium, an unusual finding. Small artery did not show any abnormality. Immunofluorescent staining of 4 glomeruli showed IgA 2+, IgG 3+, IgM 4+, C3 3+, C4 3+, C1q 3+, and fibrinogen 3+ deposition along glomerular capillaries and mesangial areas. Electron microscope showed mesangial matrix hyperplasia with insertion, basement membrane thickening, with electron dense deposit in subepithelial, endodermic and mesangial region, accompanied by epithelial foot process fusion diffusely (Figure 1). Tubular atrophy and interstitial fibrosis were also found, suggesting the diagnosis of MPGN, but the prominent infiltration of eosinophils was unusual, arousing the suspicion of secondary MPGN.

**Figure 2.**
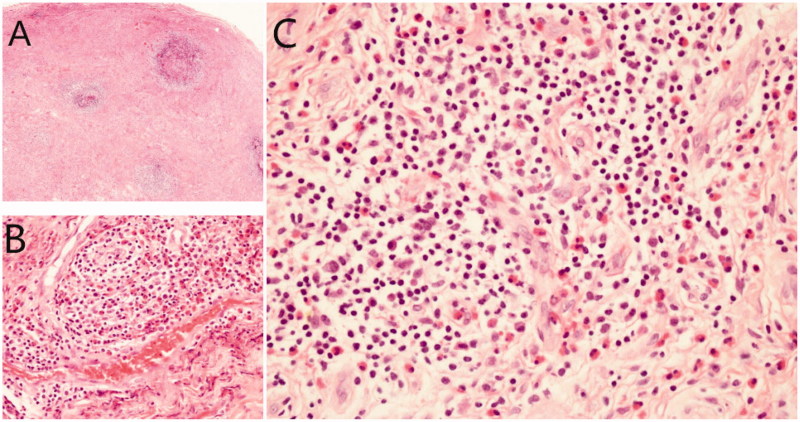
Lymph node specimen showed abundant eosinophils infiltration. (A: HE × 4; B: HE × 20; C: HE × 40).

Meanwhile, this patient underwent lymph node biopsy, which showed eosinophilic lymphoid follicular granuloma (Figure 2). Immunohistochemical staining showed positive S-100 and CD1a, but negative CD68, lysozyme, CD15, CD20, CD3, CD138, CD30, CD117, and EB virus-encoded small RNA, BCR - ABL fusion gene test was negative. A biopsy of bone marrow showed no abnormality. He was finally diagnosed as Kimura’s disease based on compatible clinical features and typical histopathological findings.

**Figure 3.**
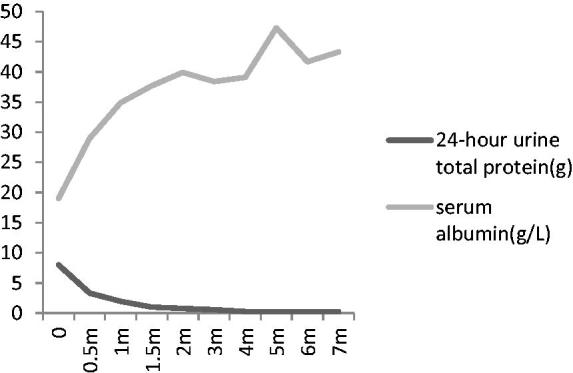
Changes of 24-h urine total protein and serum albumin according to therapy.

**Figure 4.**
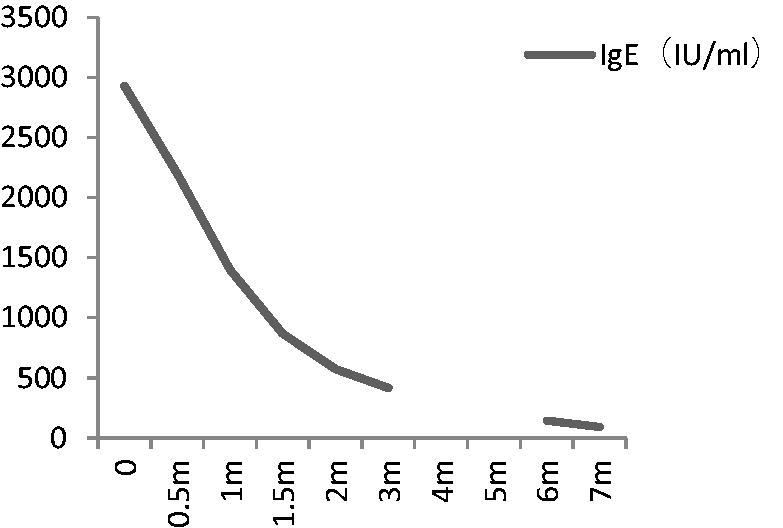
Changes in serum IgE according to therapy.

We prescribed valsartan for blood pressure control initially, with fair results (post-treatment blood pressure between 120–130/75–85 mmHg). After he received the diagnosis of Kimura’s disease, we started him on prednisone 60 mg/day (1 mg/Kg), with tapering to 55 mg/day after 2 months, followed by further reduction of 2.5 mg every 2 weeks. His bilateral post-auricular and submandibular lymphadenopathy shrank and became impalpable after the initiation of prednisone for 2 weeks. Peripheral eosinophilia subsided during the day of prednisone initiation and did not recur. 24 h urine total protein decreased gradually, accompanied by an increase in serum albumin (Figure 3). Serum IgE also decreased after treatment (Figure 4). The patient reached complete remission 3 months after prednisone treatment, a phenomenon that differs significantly from a typical case of idiopathic MPGN. 31 months later, he remained relapse-free and no adverse effect was observed.

## Discussion

Kimura’s disease is frequently characterized by the formation of subcutaneous head and neck nodules, and parotid involvement is not uncommon. Nodules can be single or multiple, one-sided or bilateral, with intact overlying skin with or without pruritis or dermatitis. Atypical nodules may be found in areas such as the upper arm [4] or the axillary region [5], but nodules can occur in any region. An important differential diagnosis of Kimura’s disease nodule is angiolymphoid hyperplasia with eosinophils, which commonly affects middle-aged women, with histopathological features of abnormal vascular proliferation, diffuse eosinophil infiltrations, and proliferative capillary endothelia with round to polygonal-shaped nuclei. Kimura’s disease, on the contrary, often involves young man of Asian origin, typically presents with elevated peripheral eosinophils and serum IgE. The most important histopathological finding of Kimura’s disease is hyperplastic follicles with germinal centers surrounded by the eosinophilic infiltrations [4,6]. Other differential diagnoses of Kimura’s disease include Hodgkin lymphoma and metastatic nodules, but these disorders can be readily identified through appropriate clinical and pathological examinations. In this case, the patient presented with subcutaneous tumor-like nodules over bilateral post-auricular and submandibular areas, which were typical sites of involvement in Kimura’s disease. Lymph node pathology showed eosinophilic lymphoid follicular granuloma, which distinguished it from other diseases with similar presentations.

This patient also had renal involvement, presenting with nephrotic syndrome, for which he underwent renal biopsy. Chen et al. [3] have analyzed clinicopathological features in 29 Chinese Kimura’s disease with renal involvement, which showed that the most common features are proteinuria, hypertension, micro hematuria with minimal change, and mesangial proliferative glomerulonephritis. However, in this case, renal biopsy showed MPGN-like features. Besides, under light microscopy, the specimen showed prominent eosinophilic infiltrations of the interstitium, and immunofluorescent staining showed positivity for all the immunoglobulin types. MPGN was typically divided into primary MPGN (also known as ‘idiopathic MPGN’) and secondary MPGN. As sophistication in analysis of biopsy material and serological methodology improved, idiopathic MPGN is definitely an ‘endangered species’, because most cases with MPGN have an underlying etiology such as infections, dysproteinemia, autoimmune disease or neoplastic disease [7]. Therapy strategy and prognosis depend on their etiology [8]. As in this case, the patient was steroid-sensitive on either the renal damage or the other involvement such as the lymph nodes and eosinophils, this mainly due to the steroid-sensitive nature of Kimura’s disease. It is widely believed that immune dysregulation is important pathogenesis of Kimura’s disease, but the exact mechanism remains unknown. Otah N et al. [9] suggest that the predominance of Th2 and Tc1 cells contributes to the pathogenesis of Kimura's disease, while others indicate that the elevation of certain cytokines play a role as well, but studies of high quality and sufficient samples are still lacking [1,10,11].

Optimal therapy for Kimura’s disease is still unclear. When subcutaneous nodules occur without renal involvement, excision may be curative [12], but some eventually develop recurrence. For those with relapse [13] or with renal involvement, systemic glucocorticoid has been suggested as a viable option. Patients with nephrotic syndrome can have disease remission through steroid use. Anecdotal reports [2,14] identified that *Tripterygium wilfordii,* leflunomide, tacrolimus, mycophenolate mofetil, or renin-angiotensin system inhibitor could be effective for treating Kimura’s disease, but limited evidence exists for their efficacy. For those with steroid-resistant status or with contraindications for using corticosteroid, irradiation may be considered, with a total dose of 20–30 Gy found to be effective [15].

## Conclusion

Kimura’s disease with nephrotic syndrome caused by secondary MPGN can be steroid-sensitive, and long term prednisone therapy is effective and safe with a low chance of relapse.
